# Zika virus and neurologic autoimmunity: the putative role of gangliosides

**DOI:** 10.1186/s12916-016-0601-y

**Published:** 2016-03-21

**Authors:** Juan-Manuel Anaya, Carolina Ramirez-Santana, Ignacio Salgado-Castaneda, Christopher Chang, Aftab Ansari, M. Eric Gershwin

**Affiliations:** Center for Autoimmune Diseases Research (CREA), Universidad del Rosario, Bogota, Colombia; Servicio de Neurología, Hospital Central de la Policía, Bogota, Colombia; Division of Rheumatology, Allergy and Clinical Immunology, University of California Davis, School of Medicine, Davis, CA USA; Department of Pathology, Emory University School of Medicine, Atlanta, GA USA

**Keywords:** Autoimmunity, Gangliosides, Guillain-Barré syndrome, Microcephaly, Zika virus

## Abstract

An increasing number of severe neurological complications associated with Zika virus (ZIKV), chiefly Guillain-Barré syndrome (GBS) and primary microcephaly, have led the World Health Organization to declare a global health emergency. Molecular mimicry between glycolipids and surface molecules of infectious agents explain most of the cases of GBS preceded by infection, while a direct toxicity of ZIKV on neural cells has been raised as the main mechanism by which ZIKV induces microcephaly. Gangliosides are crucial in brain development, and their expression correlates with neurogenesis, synaptogenesis, synaptic transmission, and cell proliferation. Targeting the autoimmune response to gangliosides may represent an underexploited opportunity to examine the increased incidence of neurological complications related to ZIKV infection.

## Background

Health authorities are on high alert over the spread of the Zika virus (ZIKV). Autochthonous cases, defined as cases through local onset of disease rather than being acquired from a different location or country and introduced into the community, have been identified in many countries in the Americas. The first cases occurred in Easter Island in February 2014 [1]. This was followed by outbreaks of autochthonous cases of ZIKV in May and October 2015 in Brazil and Colombia, respectively [1]. Since then, more than 30 countries/territories in the Americas have reported autochthonous, confirmed ZIKV infection cases [1].

Infection by ZIKV, transmitted by the Aedes mosquitos, usually ranges from completely asymptomatic cases to cases with very mild and self-limiting disease [2]. The typical symptoms include rash, fever, arthralgias, and conjunctivitis. However, in Latin America and in the South Pacific, from where it is believed the spread originated, there have been increasing reports of neurological complications attributable to ZIKV. It is the sudden increase in microcephaly and Guillain-Barré syndrome (GBS) [3–5] that prompted the World Health Organization to declare a “*public health emergency of international concern*” [3]. This type of warning is in effect a global health emergency.

The rapid spread of the epidemic has not allowed rigorous clinical studies to be conducted in Latin America [6]. However, a causal relationship between ZIKV and neurological complications is very likely [3–5]. Decisions in both medical and public health policy are based on probabilities; in this case, the factors (and cofactors) associated with neurological complications should be investigated and the risks of developing such complications defined. It is becoming clear that not all the ZIKV infected individuals will develop GBS nor will all the ZIKV-infected pregnant women deliver babies with microcephaly [7]. The identification of the risk factors associated with the development of neurological complications is the subject of present and future research studies.

The rapid spread of ZIKV in Latin America is believed to be a result of the high Aedes mosquito densities, their adaptation to urban environments, and the lack of prior immunity [2]. An important strategy to mitigate the spreading of the virus is therefore source control of the mosquito vector. Some similarities have been observed between ZIKV and dengue and chikungunya viruses, including the migratory paths taken, but it should be emphasized that there are significant differences between the clinical presentations of ZIKV infection and these other viral illnesses [8]. The limited research resources in Latin America, coupled with the increasingly apparent serious neurological complications of ZIKV infections, make a global response to controlling the epidemic even more urgent.

## Neurologic autoimmunity and ZIKV

Microcephaly literally means small head; it is a clinical finding characterized by a significant reduction in the occipital-frontal head circumference compared with age- and sex-, but sometimes not ethnically-matched controls [9]. It is classified as primary when it is detectable prior to 36 weeks of gestation and secondary in cases that develop post-partum [9]. Viral infection with agents such as cytomegalovirus in early pregnancy is one of the most common causes of primary microcephaly, and is thought to be due to a failure or reduction in the neurogenesis of neurons [9]. On the other hand, cases of microcephaly associated with either dengue or chikungunya viruses have not been proven.

GBS encompasses a number of related acute autoimmune neuropathies, although the term is also used more specifically to define patients with a peripheral polyneuropathy that usually affects all four limbs and may or may not involve cranial nerve pathology [10]. Autoimmune diseases, such as GBS, result most likely from an epigenetic modification or environmental trigger in a genetically susceptible host [11]. Both humoral and cellular immune responses against epitopes of antigens expressed by Schwann cells, myelin, or axons have been postulated to be responsible for acute autoimmune neuropathies [12]; both infectious and non-infectious triggers have been reported [13]. Two-thirds of GBS cases are preceded by symptoms of upper respiratory tract infection or gastrointestinal infections [13].

That there is a role for infection in the pathogenesis of autoimmunity has been clearly demonstrated [11, 14], but the precise mechanism by which disease develops in some individuals but not in others is still not completely clear. Infectious agents can trigger autoimmune diseases through different mechanisms, including molecular mimicry, epitope spreading, bystander activation, the production of super-antigens, and aberrant activation of the immune response [11, 15]. Reconciling the criteria for autoimmune disease definition with Koch’s postulates could provide a better understanding of the relationship between these conditions and infections [15]. However, these postulates are based on a simplistic view of autoimmune diseases because they do not take into account the multifactorial nature of autoimmunity.

Gangliosides are sialic acid-containing glycolipids found predominantly in the nervous system [16]. Antibodies that recognize gangliosides play a critical role in the pathogenesis of GBS [12, 17]. A potential mechanism is through molecular mimicry between naturally occurring glycolipids on cells and tissues of the host and surface molecules on the infectious agents. The clinical subtypes of GBS are related to the antigenic specificities of these antibodies, and the distribution of gangliosides in peripheral nervous tissues may help explain the heterogeneous clinical presentation of GBS [12, 17]. Autoantibody specificities may also be explained by the ‘binding site drift’ hypothesis, which proposes that B cells producing normally occurring anti-ganglioside antibodies (‘normal reactivity’) undergo spontaneous mutations of V genes, thereby randomly re-shaping their binding sites [18]. This phenomenon may lead to an increase in binding affinity and specificity for gangliosides, leading to the production of an inflammatory response [18].

Gangliosides, particularly GM1, GD1a, GD1b, and GT1, are especially abundant in the brain. The concentration of these compounds is about five-fold higher in grey matter than in white matter, and their expression correlates with neurogenesis, synaptogenesis, synaptic transmission, and cell proliferation [16]. It is important to note that gangliosides have been shown to be critical for brain development [19, 20].

Are gangliosides the link between ZIKV, GBS, and microcephaly? If so, why does the virus affect fetal brain development in only a small percentage of infected, pregnant women? Besides the direct neurotrophic effect of ZIKV [21, 22], the indirect effects it can induce through an autoimmune response similar to those observed in GBS (directed at glycosylation sites within the viral envelope protein) [23] or those proposed by the Gestational Neuro-Immunopathology hypothesis should be considered [24]. Thus, it is possible that during virus replication, the virus incorporates host glycolipids and/or glycoproteins expressed on the host cell membrane in a form that becomes antigenic in hosts, with selected major histocompatibility complex alleles and other genetic variants initiating an immune response that cross-reacts with similar structures expressed by neurons, for example.

## Conclusions

Studies designed to identify the targets of the autoimmune response to molecules such as gangliosides may represent an underexploited opportunity to examine the increased incidence of neurological complications related to ZIKV infection. An understanding of the role of genetics, epigenetics, and the environment in the pathogenesis of autoimmunity would help identify susceptible individuals, tailor effective personalized treatment strategies, and minimize the adverse effects of these diseases.

## Abbreviations

GBS: Guillain-Barré syndrome
ZIKV: Zika virus
